# The Condition of the Meniscus and Cartilage of the Injured Knee on Preoperative Magnetic Resonance Imaging Is a Prognostic Factor Affecting Postoperative Outcomes Following Knee Cartilage Restoration Surgery

**DOI:** 10.1016/j.asmr.2024.100973

**Published:** 2024-07-14

**Authors:** Dai Sato, Rawee Manatrakul, Chotigar Ngarmsrikam, Brian T. Feeley, C. Benjamin Ma, Thomas M. Link, Drew A. Lansdown

**Affiliations:** a Department of Orthopedic Surgery, Sports Medicine & Shoulder Surgery, University of California, San Francisco, San Francisco, California, U.S.A.; b Department of Orthopedic Surgery, Hokkaido University Graduate School of Medicine, Sapporo, Japan; c Musculoskeletal and Quantitative Imaging Research, Department of Radiology and Biomedical Imaging, University of California, San Francisco, San Francisco, California, U.S.A.; d Department of Radiology, Faculty of Medicine Ramathibodi Hospital, Mahidol University, Bangkok, Thailand

## Abstract

**Purpose:**

To evaluate the relationship between preoperative whole-joint imaging evaluation of the knee with patient-reported outcome (PRO) measures after cartilage restoration surgery (mosaicplasty, osteochondral allograft transplantation, matrix autologous chondrocyte implantation).

**Methods:**

We retrospectively evaluated patients who underwent knee articular cartilage restoration at our institution from 2014 to 2020. The patients’ knee magnetic resonance imaging (MRI) was evaluated with the Whole-Organ Magnetic Resonance Imaging Score (WORMS) and semiquantitative synovial inflammation imaging biomarkers of the preoperative MRI. To assess PRO score, Lysholm score and Knee injury and Osteoarthritis Outcome Score were completed at a minimum 2-year follow-up. Statistical analysis was performed using the Spearman rank test to obtain correlation values for WORMS score and PRO score for each survey.

**Results:**

Forty patients were enrolled in this study. The average age at baseline was 34.5 years. The average body mass index was 28.2, and 26 of 40 were men (age range, 20-58 years). The maximum preoperative WORMS score was significantly correlated with the postoperative Lysholm score (*r* = –0.52, *P* = .0013). The WORMS Meniscus and Cartilage subscales were significantly correlated with the Lysholm score (*r* = –0.36, *P* = .024 and *r* = –0.37, *P* = .021, respectively). The maximum WORMS score was significantly correlated with the Knee injury and Osteoarthritis Outcome Score daily living and sports/recreation subscores (*r* = –0.47, *P* = .0023 and *r* = –0.42, *P* = .0077, respectively). Semiquantitative synovial inflammation imaging biomarkers were not significantly correlated with PRO scores.

**Conclusions:**

Increasing preoperative degenerative change in the knee, as evidenced by a higher WORMS on preoperative MRI, was associated with inferior patient-reported outcomes at a minimum of 2 years after cartilage restoration surgery (mosaicplasty, osteochondral allograft transplantation, matrix autologous chondrocyte implantation). Semiquantitative scoring of the whole joint on preoperative MRI may allow for improved counseling regarding expected benefit for patients after cartilage restoration surgery.

**Level of Evidence:**

Level IV, prognostic case series.

Multiple surgical options for knee cartilage restoration allow for favorable patient outcomes.^1^, ^2^, ^3^, ^4^, ^5^, ^6^ The principal function of knee articular cartilage is to provide a smooth, lubricated surface for articulation and to facilitate the transmission of loads with a low frictional coefficient.^7^^,^^8^ Cartilage defects may occur as a focal, isolated lesion or can be observed concurrently with other degenerative changes at the knee joint. Although there is a wide spectrum of potential cartilage pathology and various procedures to restore them, little is known about which patients benefit the most from undergoing these procedures. Having rigorous predictors of patient-related outcomes, including pain and function, would help to better guide recommendations for cartilage restoration therapy.

The Whole-Organ Magnetic Resonance Imaging Score (WORMS) is a semiquantitative method for evaluation of abnormalities of the entire knee joint.^9^^,^^10^ This scoring system provides a reproducible semiquantitative grading to evaluate cartilage and meniscus condition as well as bone marrow abnormalities in the compartments of the knee on standard magnetic resonance imaging (MRI) and has been shown to have excellent intra- and inter-reader reproducibility.^10^ A previous study has shown that detailed evaluation of the meniscus, cartilage, and bone marrow lesions allows for prediction of continued progression of degenerative changes in the knee.^11^ Providing an overall assessment of MRI-based tissue-specific structural joint health may potentially allow guidance concerning expected clinical outcomes.

The purpose of this study change was to evaluate the relationship between preoperative whole-joint imaging evaluation of the knee with patient-reported outcome (PRO) measures after cartilage restoration surgery (mosaicplasty, osteochondral allograft transplantation [OCA], matrix autologous chondrocyte implantation [MACI]). We hypothesized that patients with worse preoperative knee cartilage or meniscus lesion on MRI will have worse postoperative outcome survey scores at a minimum 2-year follow-up after cartilage restoration.

## Methods

Patients who underwent knee articular cartilage restoration (mosaicplasty, OCA, MACI) in the tibiofemoral or patellofemoral knee joint at our tertiary-care institution (University of California, San Francisco) from 2014 to 2020 were retrospectively identified. Informed consent from each patient was obtained electronically, and institutional review board approval was obtained for this study.

The inclusion criteria of this study were ages 18 to 60 years, articular cartilage restoration (mosaicplasty, OCA, and autologous chondrocyte implantation [ACI]) in the tibiofemoral or patellofemoral knee joint, a minimum of 2-year postoperative follow-up, and preoperative knee MRI available for review. The exclusion criteria of this study were prior ipsilateral knee surgery except for MACI biopsy, associated ipsilateral knee ligamentous injury that required surgical treatment, history of additional injury requiring surgery during the follow-up window, history of revision surgery, and conversion to total knee arthroplasty.

Demographics of these identified patients were recorded, including patient sex, age at time of surgery, and body mass index (BMI).

### Surgical Procedures of Cartilage Restoration Surgery

All surgeries were performed by 3 sports medicine fellowship-trained surgeons (B.T.F., C.B.M., D.A.L.). Patients were retrospectively identified, recruited, and enrolled in the study. In all cases, a complete arthroscopic evaluation of all the compartments of the knee was conducted to confirm the size and depth of the lesion and to address any concurrent intraarticular pathology. (1) Mosaicplasty was performed using an osteochondral autograft transfer system (OATS; Arthrex).^6^ (2) In case of OCA, an authorized tissue bank supplied the allografts and performed all the preoperative graft processing.^3^, ^4^, ^5^ After sizing the articular defect, the guide pin was placed perpendicularly. The socket was reamed to a depth of 6 to 10 mm. The fresh allograft was fashioned to match this socket. Finally, it was implanted into place. (3) In case of ACI, a standard, 2-stage surgical MACI technique was used as previously described in detail.^12^, ^13^, ^14^, ^15^ A cartilage biopsy was harvested from a nonweightbearing area of the intercondylar notch with a ring curette,^16^ and chondrocytes were isolated (Vericel), cultured, and seeded onto a porcine collagen membrane (MACI; Vericel). Standardized MACI implantation was performed using mini knee arthrotomy. All patients underwent a similar standardized postoperative protocol that included nonweightbearing for 6 weeks in a hinged knee brace and physical therapy for 6 months to 1 year.

### Clinical Evaluation/Outcome Scores

PROs comprised Lysholm score,^17^ the patient-reported outcome measurement information system physical function computer adaptive test (PROMIS PF-CAT),^18^ Knee injury and Osteoarthritis Outcome Score (KOOS),^19^ and visual analog scale (VAS) score.

### MRI Semiquantitative Grading

Preoperative knee baseline magnetic resonance studies were obtained at 3T with a standard protocol that included sagittal, coronal, and axial fat saturated intermediate weighted fast spin echo (FSE) sequences (field of view, 140; slice thickness, 3 mm; repetition time/echo time, 3,200/30 ms), coronal T1-weighted and sagittal proton density weighted FSE sequences (field of view, 140; slice thickness, 3 mm; repetition time/echo time, 2,700/20 ms). All studies were assessed by 2 musculoskeletal radiologists (R.M., C.N.; 7 years of experience and 11 years of experience) blinded to subject characteristics and under supervision of a board-certified musculoskeletal radiologist (T.M.L.) who served as an adjudicator for any disagreements in review (24 years of experience). The modified WORMS is a semiquantitative score that evaluates cartilage, bone marrow, and meniscus abnormalities in 6 compartments of the knee and was used in multiple studies with excellent reproducibility.^20^, ^21^, ^22^, ^23^ Each compartment and the entire knee have a tissue-specific (e.g., cartilage and meniscus) maximum score in addition to a whole-knee WORMS score. WORMS summation scores were calculated for each lesion type (score range: cartilage, 0-36; meniscus, 0-24; ligament and tendon, 0-21; bone marrow edema-like lesions [BMELL], 0-18; subchondral cyst-like lesions, 0-18; joint effusion, 0-3), for each examination. The osteophyte subscale was excluded in our cohort, as this MRI has limitations in assessing osteophytes.^24^ WORMS score was described in detail. Cartilage abnormalities were scored using an 8-point scale: 0 = normal thickness and signal; 1 = normal thickness but abnormal signal on fluid-sensitive sequences; 2.0 = partial-thickness focal defect <1 cm in greatest width; 2.5 = full-thickness focal defect <1 cm in greatest width; 3 = multiple areas of partial-thickness (grade 2.0) defects intermixed with areas of normal thickness, or a grade 2.0 defect wider than 1 cm but <75% of the region; 4 = diffuse (≥75% of the region) partial-thickness loss; 5 = multiple areas of full-thickness loss (grade 2.5), or a grade 2.5 lesion wider than 1 cm but <75% of the region; and 6 = diffuse (≥75% of the region) full-thickness loss. Alterations in meniscal morphology were assessed separately in 6 regions (medial and lateral: anterior, body, posterior) using a 4-level scale (0, normal; 1, intrasubstance abnormalities; 2, nondisplaced tear; 3, displaced or complex tear; 4, complete destruction/maceration). Meniscal extrusion was graded as follows: 0 (none) and 1 (meniscal extrusion of more than 3 mm beyond the tibia plateau). Subarticular bone marrow abnormalities were defined as poorly marginated areas of increased signal intensity in the normal subchondral and epiphyseal bone marrow on fat-suppressed fluid-sensitive FSE sequences. This feature was graded from 0 to 3 based on the extent of regional involvement: 0 = none, 1 = ≤25% of the region, 2 = 25% to 50% of the region, and 3 = >50% of the region. Ligaments and joint effusion were evaluated using a 4-point scale from 0 to 3 (0 = no lesion, 1 = grade 1 sprain [signal changes around ligament], 2 = grade 2 sprain [partial tear], 3 = grade 3 sprain [complete tear] for ligaments; 0 = normal, 1 = <33% of maximum potential distention, 2 = 33%-66% of maximum potential distention, 3 = >66% of maximum potential distention for joint effusion). Based on the magnetic resonance findings, a knee was defined as abnormal if a WORMS value of ≥1 was found. Semiquantitative synovial inflammation imaging biomarkers as described in previous publications^25^, ^26^, ^27^, ^28^ were also obtained, including effusion synovitis, size and intensity of infrapatellar fat pad signal abnormality (Hoffa synovitis), and synovial proliferation score. Semiquantitative synovial inflammation imaging biomarkers were described in detail. First, we graded the extent of effusion synovitis by measuring the maximum anteroposterior diameter of the suprapatellar recess on midline sagittal images, according to the Anterior Cruciate Ligament Osteoarthritis Score (ACLOAS).^28^ The grading ranged from 0 to 3, based on the degree of capsular distension: grade 0 corresponded to an anteroposterior diameter of <2 mm, grade 1 to a diameter of ≥2 to <5 mm, grade 2 to a diameter of ≥5 to <10 mm, and grade 3 to a diameter of ≥10 mm. Second, effusion synovitis was also graded on axial images using the MRI Osteoarthritis Knee Score (MOAKS),^26^ with a 4-point scale: 0 for a physiologic amount of fluid; 1 for a small, continuous extension into the retropatellar space; 2 for medium, indicating slight convexity of the suprapatellar bursa; and 3 for large, indicating evident capsular distention. Third and fourth, we assessed the size and highest signal intensity of Hoffa’s or the infrapatellar fat pad (IPFP) abnormalities on sagittal fat-suppressed images.^27^ The size of IPFP signal abnormalities was categorized as follows: grade 0 = no abnormality, grade 1 = abnormalities in ≤33% of the region, grade 2 = abnormalities in 34% to 66% of the region, and grade 3 = abnormalities in ≥66% of the region. Signal intensity was graded as follows: grade 0 = none, grade 1 = mild (lower than cartilage), grade 2 = moderate (equal to or higher than cartilage but lower than fluid), and grade 3 = severe (equal to fluid). Fifth, we evaluated the presence and severity of synovial proliferations in the knee if the effusion synovitis score was ≥1 by either ACLOAS or MOAKS methods, focusing on the suprapatellar recess and other visible areas.^25^ Grade 1 indicated smooth synovium without visible proliferation or bands; grade 2 indicated mild synovial irregularity, either focal or diffuse, with some bands or small bodies; and grade 3 indicated extensive synovial thickening with irregular villonodular proliferation. This same synovial proliferation scoring was applied to knees with a popliteal cyst.

### Inter-reader Reproducibility

Reproducibility for composite WORMS gradings and MRI synovial inflammatory scores was assessed in all patients. All gradings were performed by 2 musculoskeletal radiologists (R.M., C.N.) for inter-reader reproducibility.

### Statistical Analysis

All data were presented as the mean ± standard deviation. Spearman rank tests were performed to determine the associations between WORMS score and each of the PRO metrics. Correlation strengths were defined as high (≥0.7), high-moderate (0.61-0.69), moderate (0.4-0.6), moderate-weak (0.31-0.39), and weak (≤0.3).^29^ Inter-reader reproducibility measurements for WORMS and MRI synovial inflammation markers were tested by weighted κ values. According to Landis and Koch,^30^ a κ value of less than 0.00 indicates poor agreement; a value of 0.00 to 0.20 slight agreement, a value of 0.21 to 0.40 fair agreement, a value of 0.41 to 0.60 moderate agreement, a value of 0.61 to 0.80 substantial agreement, and a value of 0.81 to 1.00 almost perfect agreement. Statistical significance was defined as *P* < .05.

## Results

### Patient Demographics

**Fig 1:**
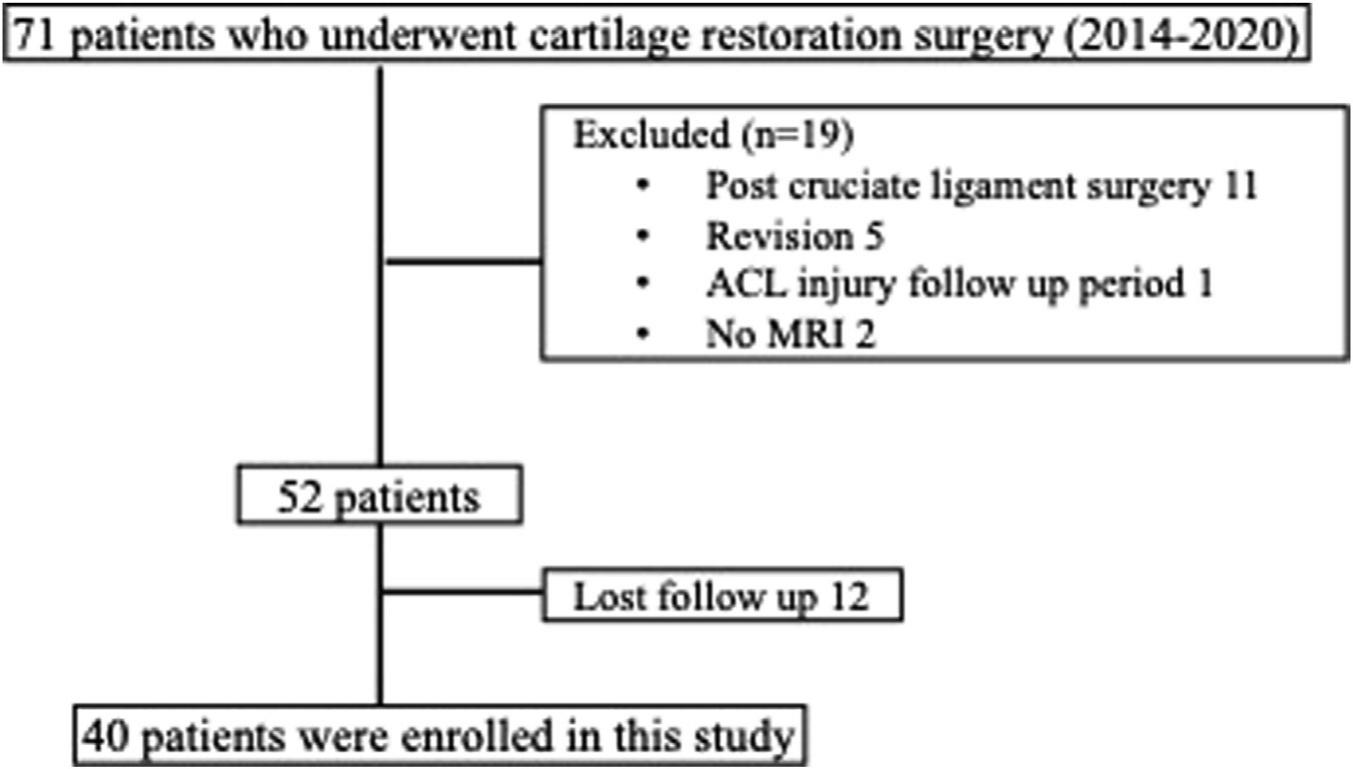
Study design. (ACL, anterior cruciate ligament; MRI, magnetic resonance imaging.)

A total of 71 patients were identified for potential inclusion in this study. Nineteen patients were excluded and 12 were lost during follow-up (Fig 1). Forty patients (26 men and 14 women) with a mean age of 34.4 years (range, 20-58 years) were included in this evaluation (Table 1). The mean BMI was 28.3. Defect location was most commonly in the medial femoral condyle (n = 18), followed by the lateral femoral condyle (n = 14), patella (n = 4), and trochlea (n = 4). Sixteen patients underwent treatment with mosaicplasty, 16 were treated with osteochondral allograft, and 8 patients received MACI.

**Table 1:** Descriptive Statistics of Baseline Demographic, Surgical Parameters, and 2-Year Outcome Variables in 40 Patients

	Value	Range
Baseline characteristics		
Age, y	34.4 ± 9.6	20-58
Male	26 (65)	
BMI	28.3 ± 5.6	19.1 – 41.6
Kellgren-Lawrence grade		
0	15 (37.5)	
1	19 (47.5)	
2	6 (15.0)	
3	0 (0)	
Surgical characteristics		
Cartilage defect area		
Medial femoral condyle	18 (45)	
Lateral femoral condyle	14 (35)	
Patella femoral joint		
Patella	4 (10)	
Trochlea	4 (10)	
Type of surgery		
Mosaicplasty	16 (40)	
OCA	16 (40)	
MACI	8 (20)	
Follow-up period, y	3.6 ± 1.4	2.0-6.3

NOTE. Data are reported as mean ± standard deviation or number (%) unless otherwise indicated.

BMI, body mass index; MACI, matrix autologous chondrocyte implantation; OCA, osteochondral allograft transplantation.

### Preoperative MRI Findings

**Fig 2:**
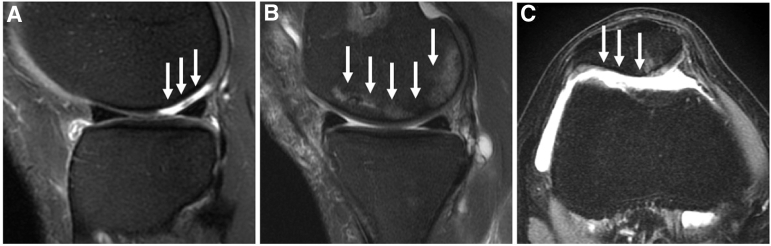
Magnetic resonance imaging findings in patients with high Whole-Organ Magnetic Resonance Imaging Score. (A) Sagittal T2-weighted fast spin echo (FSE) image shows grade 5 cartilage lesion (white arrows) in the lateral femoral condyle. (B) Sagittal T2-weighted FSE image shows grade 3 bone marrow edema-like lesion (white arrows) in the lateral femoral condyle. (C) Axial T2-weighted FSE image shows grade 5 cartilage lesion in the patella (white arrows) with subjacent grade 2 (white arrowheads).

The preoperative MRI findings are shown in Table 2. The average WORMS total and MRI synovial inflammatory markers total were 14.1 ± 6.96 and 5.95 ± 2.74, respectively. The images of a patient with the worst WORMS are shown in Figure 2.

**Table 2:** Preoperative MRI Findings

	Mean ± Standard Deviation
WORMS	
Meniscus lesions (0-24)	2.55 ± 4.03
Cartilage lesions (0-24)	7.54 ± 2.88
Ligament abnormalities (0-21)	1.120 ± 1.87
Bone marrow (BMELL) (0-18)	2.70 ± 1.98
Subchondral cyst (0-18)	0.25 ± 0.81
Effusion (0-3)	1.105 ± 0.98
Maximum score (0-108)	14.1 ± 6.96
MRI synovial inflammatory markers	
Extent of effusion synovitis according to ACLOAS (0-3)	1.08 ± 1.02
Effusion synovitis according to MOAKS (0-3)	1.48 ± 0.64
Hoffa’s signal (0-3)	1.23 ± 0.92
Hoffa’s size (0-3)	0.83 ± 0.59
Synovial proliferation score (0-3)	1.35 ± 0.70
Total (0-15)	5.95 ± 2.74

ACLOAS, Anterior Cruciate Ligament Osteoarthritis Score; BMELL, bone marrow edema-like lesion; MOAKS, MRI Osteoarthritis Knee Score; MRI, magnetic resonance imaging; WORMS, Whole-Organ Magnetic Resonance Imaging Score.

### Postoperative PRO Scores

The average Lysholm score, PROMIS PF-CAT, VAS pain score, and KOOS total score were 80.0 ± 16.3, 49.5 ± 7.7, 1.5 ± 1.4, and 80.2 ± 17.7, respectively. The average KOOS symptoms + stiffness, pain, function (daily living), function (sports and recreational activities), and quality of life were 80.8 ± 20.0, 82.5 ± 20.2, 90.6 ± 15.9, 68.6 ± 23.7, and 61.3 ± 26.4, respectively. There were no significant differences between mosaicplasty, OCA, and ACI groups.

### Correlation of Preoperative WORMS With Postoperative PRO scores

The maximum WORMS score was significantly correlated with the Lysholm score (*r* = –0.52; 95% confidence interval [CI], –0.72 to –0.21), PROMIS PF-CAT (*r* = –0.39; 95% CI, –0.64 to –0.068), and VAS pain score (*r* = 0.47; 95% CI, 0.16 to 0.69) (Table 3). The WORMS meniscus subscale was significantly correlated with the Lysholm score (*r* = –0.36; 95% CI, –0.62 to –0.041), the WORMS cartilage subscale was significantly correlated with the Lysholm score (*r* = –0.37; 95% CI, –0.62 to –0.051) and the VAS pain score (*r* = 0.36; 95% CI, 0.029 to 0.61), and the WORMS bone marrow edema subscale was significantly correlated with the VAS pain score (*r* = 0.36; 95% CI, 0.033 to 0.62). The maximum WORMS score was significantly correlated with the KOOS function/daily living and sports/recreation subscore (*r* = –0.47; 95% CI, –0.69 to –0.17 and *r* = –0.42; 95% CI, –0.66 to –0.11) (Table 4). The WORMS cartilage subscale was significantly correlated with the KOOS function/daily living and the sports/recreation subscore (*r* = –0.40; 95% CI, –0.64 to –0.10 and *r* = –0.36; 95% CI, –0.61 to –0.047), respectively. The WORMS effusion subscale was significantly correlated with the KOOS symptoms (*r* = 0.35; 95% CI, 0.032 to 0.56).

**Table 3:** Statistical Analysis: Spearman Correlation Between PRO Score and WORMS

	Lysholm Score		PROMIS PF-CAT		VAS Pain Score	
	*r*	95% CI	*r*	95% CI	*r*	95% CI
WORMS						
Maximum score	**–0.52**∗	**–0.72 to** –**0.21**	**–0.39**∗	**–0.64 to** –**0.068**	**0.47**∗	**0.16 to 0.69**
Meniscus lesions	**–0.36**∗	**–0.62 to** –**0.041**	–0.29	–0.53 to 0.089	0.20	–0.14 to 0.50
Ligament abnormalities	–0.017	–0.49 to 0.13	0.0089	–0.25 to 0.41	0.26	–0.080 to 0.55
Cartilage defects	**–0.37**∗	**–0.62 to** –**0.051**	–0.13	–0.44 to 0.20	**0.36**∗	**0.029 to 0.61**
Bone marrow (BMELL)	–0.20	–0.49 to 0.13	0.078	–0.26 to 0.40	**0.36**∗	**0.033 to 0.62**
Subchondral cysts	–0.30	–0.57 to 0.027	–0.12	–0.44 to 0.23	0.26	–0.078 to 0.55
Effusion	0.15	–0.18 to 0.45	0.32	–0.014 to 0.59	–0.10	–0.42 to 0.23

BMELL, bone marrow edema-like lesion; CI, confidence interval; PRO, patient-reported outcome; PROMIS PF-CAT, patient-reported outcome measurement information system physical function computer adaptive test; VAS, visual analog scale; WORMS, Whole-Organ Magnetic Resonance Imaging Score.

∗ *P* < .05.

**Table 4:** Statistical Analysis: Spearman Correlation Between KOOS and WORMS

	KOOS Total		KOOS Symptoms and Stiffness		KOOS Pain		KOOS Function, Daily Living		KOOS, Sports/Recreation		KOOS QOL	
	*r*	95% CI	*r*	95% CI	*r*	95% CI	*r*	95% CI	*r*	95% CI	*r*	95% CI
WORMS												
Maximum score	–0.26	–0.42 to 0.19	–0.20	–0.42 to 0.22	–0.27	–0.58 to 0.021	**–0.47**∗	**–0.69 to** –**0.17**	**–0.42**∗	**–0.66 to** –**0.11**	–0.076	–0.39 to 0.25
Meniscus lesions	–0.10	–0.50 to 0.09	–0.18	–0.39 to 0.24	–0.069	–0.38 to 0.26	–0.23	–0.51 to 0.09	–0.11	–0.42 to 0.21	–0.042	–0.36 to 0.28
Ligament abnormalities	0.14	–0.35 to 0.27	0.15	–0.24 to 0.40	–0.056	–0.37 to 0.27	–0.16	–0.43 to 0.16	–0.15	–0.49 to 0.17	0.014	–0.31 to 0.33
Cartilage defects	–0.11	–0.33 to 0.29	–0.04	–0.34 to 0.29	–0.22	–0.51 to 0.10	**–0.40**∗	**–0.64 to** –**0.10**	**–0.36**∗	**–0.61 to** –**0.047**	0.053	–0.27 to 0.37
Bone marrow (BMELL)	–0.16	–0.20 to 0.41	–0.13	–0.25 to 0.33	–0.19	–0.48 to 0.13	–0.18	–0.48 to 0.14	–0.11	–0.42 to 0.21	0.11	–0.21 to 0.42
Subchondral cyst	–0.30	–0.42 to 0.19	–0.20	–0.56 to 0.02	–0.25	–0.58 to 0.0059	–0.15	–0.45 to 0.17	–0.19	–0.47 to 0.14	–0.073	–0.38 to 0.25
Effusion	–0.012	–0.33 to 0.31	**0.35**∗	**0.032 to 0.56**	0.076	–0.25 to 0.39	0.12	–0.20 to 0.43	0.16	–0.17 to 0.46	–0.064	–0.37 to 0.25

BMELL, bone marrow edema-like lesion; CI, confidence interval; KOOS, Knee injury and Osteoarthritis Outcome Score; QOL, quality of life; WORMS, Whole-Organ Magnetic Resonance Imaging Score.

∗ *P* < .05.

### Correlation of MRI Synovial Inflammatory Markers With PRO Scores

There was no significant correlation between MRI synovial inflammatory markers and Lysholm score, PROMIS PF-CAT, and VAS pain score. The total MRI synovial inflammatory markers were significantly correlated with the KOOS symptoms subscore (*r* = 0.33, *P* = .037), and there was no significant correlation between MRI synovial inflammatory markers and KOOS total other subscores.

### Reproducibility of Clinical Readings

Weighted Cohen’s κ values were calculated for each score separately. Inter-reader agreement/κ values were 0.99, 1.00, 1.00, 1.00, 1.00, and 0.94 for meniscus, cartilage, ligament lesions, BMELL, subchondral bone cyst, and effusion, respectively. Inter-reader agreement/κ values were 0.97 and 0.94 for effusion synovitis using ACLOAS and MOAKS methods, respectively; 0.93 and 0.93 for IPFP signal intensity and size, respectively; and 0.77 for synovial proliferation score of the knee. These findings demonstrated moderate inter-reader agreement for the synovial proliferation score and almost perfect agreement for the WORMS and other MRI synovial inflammation abnormalities.

## Discussion

In this study, we observed that increasing preoperative degenerative change in the knee, as evidenced by a higher WORMS on preoperative MRI, was associated with inferior patient-reported outcomes at a mean 3.6 years after cartilage restoration surgery (mosaicplasty, OCA, ACI). While knee cartilage restoration surgery has demonstrated good clinical efficacy for the repair of articular cartilage defects in the knee,^1^, ^2^, ^3^, ^4^, ^5^, ^6^ we understand little about the contribution of known influential preoperative factors from imaging to postoperative outcome. Of importance to the correlated factors of PRO score after knee cartilage restoration surgery, WORMS meniscus and cartilage subscales appear to be most significant in their association with the eventual subjective outcomes such as pain and function.

Previous research has demonstrated that knee cartilage restoration surgery allows for long-term success and potentially delays progression of osteoarthritis for larger chondral defects.^31^^,^^32^ Although patient demographic factors such as age and BMI are known risk factors for poor outcomes after knee cartilage restoration surgery,^33^, ^34^, ^35^, ^36^ little is known about the relationship between preoperative MRI grading and PRO scores after knee cartilage restoration surgery. Kreuz et al.^32^ reported on a correlation between radiographic changes (joint space narrowing) and clinical symptoms, although the relationship between preoperative MRI-based findings and eventual surgical outcome has not been defined previously.

The WORMS was designed and introduced to semiquantitatively assess the overall structural abnormalities of the injured knee on a standard MRI.^10^ WORMS gradings were used to assess cartilage, meniscus, ligamentous, and bone marrow abnormalities of the knee joint. As previously described,^37^, ^38^, ^39^, ^40^ the WORMS offers a very detailed assessment of the knee joint and also has a high inter-rater reliability. In our study, our reproducibility values were also very high, giving further support that this scoring system can be readily defined by trained observers.

This study outlined that preoperative MRI grading was significantly correlated with postoperative PRO scores. The strongest relationships were observed with the WORMS meniscus and cartilage subscales and their correlations with Lysholm score and KOOS sports/recreation subscore. This analysis may provide a potential screening tool for surgeons to better assess which patients may benefit from cartilage restoration surgery. Importantly, cartilage restoration surgery is both costly to the health care system and requires an extensive period of rehabilitation. Defining characteristics that may help predict an eventual outcome can help in determining which patients may benefit from treatment and allow for preoperative counseling regarding likelihood of success with joint preservation surgery. We believe these variables are important to study in future larger prospective cohorts as prognostic factors in the setting of cartilage restoration surgery. The status of the meniscus, especially, may be key in optimizing outcomes after cartilage restoration.

The WORMS cartilage subscale was also correlated with PRO scores. The WORMS subscale not only represents the size of the treated cartilage defect but also includes information on the overall cartilage condition, subchondral bone, depth of involvement, and the noninvolved articular cartilage in the knee. Given the associations observed in this cohort, we speculate the WORMS cartilage subscale may provide an extensive and comprehensive evaluation of the articular cartilage. It should be noted that there was significant correlation between WORMS subscale BMELL and postoperative VAS pain score. Previous studies showed that there was no association between BMELL and pain,^41^, ^42^, ^43^ while other studies found significant correlations.^44^ A meta-analysis by Yusuf et al.^45^ found moderate evidence for correlation of knee pain and BMELLs. Since the pain is believed to result from fibrovascular tissue and sensory nerve in growth in the area of the BMELL,^46^ worse WORMS BMELL subscale might be potential prognostic factor of pain.

We observed a positive correlation between MRI synovial inflammatory markers and KOOS symptoms subscale, indicating that patients with more significant synovial inflammation on preoperative MRI showed improved KOOS symptoms subscores at final follow-up. As stated in a systematic review of the predictive value of MRI biomarkers in osteoarthritis,^47^ synovial inflammation can promote cartilage degeneration by secreting catabolic and proinflammatory mediators. Some studies^48^, ^49^, ^50^ have reported the evidence of an association between synovitis (including effusion) and knee symptoms measured by the several PRO scores in patients with knee osteoarthritis. In contrast to these reports, studies also have observed that synovitis has been related not only to knee pain but also to knee joint function.^51^^,^^52^ The reasons for this disagreement between our results and previous ones are unclear. This observation may indicate those patients who have more symptomatic or significant cartilage defects, resulting in greater degrees of synovitis, or may highlight patients with the most potential for improvement after treatment of their cartilage defects. Future work should help define the effect of synovitis on PRO score, and it may have the greatest benefit to predict outcomes for them. The presence of synovitis on preoperative imaging may serve as a marker for those patients who will benefit most from cartilage restoration surgery.

### Limitations

This study has some limitations. First, it must be emphasized that the number of patients could have limited our study results. We are not able to control for all of the potential factors, including age, sex, BMI, race, level of activity, and location of defect with this sample size. A second limitation is that we did not collect preoperative PRO scores, which prevented us from obtaining detailed information on the patients’ condition before surgery. As a result, we were unable to detect the degree of recovery from before to after surgery and objectively assessing treatment effectiveness. This represents a relevant bias in the current analysis. Finally, multiple different methods for cartilage restoration surgery were used, which could have differential effects on eventual outcomes. Additionally, multiple compartments were included in the analysis. Different compartments in the knee have different loading patterns that may also adversely affect outcomes.

## Conclusions

Increasing preoperative degenerative change in the knee, as evidenced by a higher WORMS on preoperative MRI, was associated with inferior patient-reported outcomes at a minimum 2 years after cartilage restoration surgery (mosaicplasty, OCA, MACI). Semiquantitative scoring of the whole joint on preoperative MRI may allow for improved counseling regarding expected benefit for patients after cartilage restoration surgery.

## Disclosures

The authors declare the following financial interests/personal relationships which may be considered as potential competing interests: B.T.F. is a consultant or advisor for Stryker and the California Institute for Regenerative Medicine and has received funding grants from the 10.13039/100000002National Institutes of Health. C.B.M. is a consultant or advisor for Zimmer Biomet, CONMED, Stryker, and Aesculap AG. D.A.L. is a consultant or advisor for Vericel Corporation and AlloSource. All other authors (D.S., R.M., C.N., T.M.L.) declare that they have no known competing financial interests or personal relationships that could have appeared to influence the work reported in this paper.
